# Identity-Driven Differences in Stakeholder Concerns about Hunting Wolves

**DOI:** 10.1371/journal.pone.0114460

**Published:** 2014-12-02

**Authors:** Michelle L. Lute, Adam Bump, Meredith L. Gore

**Affiliations:** 1 Michigan State University, Department of Fisheries and Wildlife, East Lansing, Michigan, United States of America; 2 Michigan Department of Natural Resources, Wildlife Division, Lansing, Michigan, United States of America; 3 Michigan State University, Department of Fisheries and Wildlife, School of Criminal Justice, East Lansing, Michigan, United States of America; University of Tasmania, Australia

## Abstract

Whereas past wolf management in the United States was restricted to recovery, managers must now contend with publicly contentious post-recovery issues including regulated hunting seasons. Understanding stakeholder concerns associated with hunting can inform stakeholder engagement, communication, and policy development and evaluation. Social identity theory (SIT) has been used to understand how groups interact, why they conflict, and how collaboration may be achieved. Applying SIT to stakeholder conflicts about wolf hunting may help delineate groups according to their concern about, support for or opposition to the policy choice of hunting wolves. Our objective was to assess concerns about hunting as a tool to resolve conflict in Michigan, using SIT as a framework. We used a mixed-modal sampling approach (e.g., paper, Internet) with wolf hunting-related public meeting participants in March 2013. Survey questions focused on 12 concerns previously identified as associated with hunting as a management tool to resolve conflict. Respondents (n  =  666) cared greatly about wolves but were divided over hunting wolves. Wolf conflicts, use of science in policy decisions, and maintaining a wolf population were the highest ranked concerns. Principle components analysis reduced concerns into three factors that explained 50.7% of total variance; concerns crystallized over justifications for hunting. General linear models revealed a lack of geographic influence on care, fear and support for hunting related to wolves. These findings challenge assumptions about regional differences and suggest a strong role for social identity in driving dichotomized public perceptions in wildlife management.

## Introduction

Effective decision-making in wildlife management may be inhibited by conflict between and among stakeholders, especially when management decisions or actions are controversial [1], [2]. Polarization, or an “us versus them” mentality, may manifest as stakeholders organize into groups associated with differing opinions about how to manage wildlife that pose problems for humans [3]. Negative social and political repercussions associated with these “intergroup” conflicts over human-wildlife conflict (HWC) may include disenfranchisement of less powerful or minority stakeholders, non-compliance with harvest regulations or power struggles for control of natural resources [4]–[6]. Although some conflict can be useful for driving needed change, resolving negative consequences of conflict is key for effective and efficient decision-making regarding management of human-wildlife conflict [2], [7], [8]. Herein, we apply principles from social psychology to understand the “us versus them” dynamic that has manifested over hunting wolves in Michigan. We sought to document attitudinal diversity among identity groups identified in prior work [3] and to further delineate these groups according to their concerns about the policy choice of hunting wolves as a management tool as well as care for and fear of wolves. In characterizing specific stakeholder concerns and exploring associated social identities underlying these concerns, our aim is to assess the extent to which SIT may help to improve HWC management.

One dominant paradigm for reducing conflicts among stakeholders over HWC is stakeholder engagement [9], [10]. State wildlife agencies, nongovernmental organizations, and other groups engage different stakeholder groups in participatory decision-making processes with the intention of, among other things, increasing buy-in for decision outcomes [11]. Sociodemographic variables such as occupation, organizational membership, political ideology or residence (i.e., urban, rural) are commonly used to segment publics and determine representation [12], [13] for these participatory activities. Successful participatory-based decision-making processes in HWC management have been well documented [14]–[16]. Sometimes, participatory decision-making processes may not adequately uncover the underlying complexity of intergroup conflicts and the root of conflict remains obscured. This is problematic because in such instances, participatory decision-making processes may fail to achieve objectives and result in ineffective policy or inefficient use of resources [10], [17].

Psychology's social identity theory (SIT) posits that perceptions of unequal power help drive intergroup competition and bias individuals against competing groups with different ideologies [18], [19]. SIT may provide a lens through which to consider the causes and consequences of intergroup conflict, including why stakeholders interact and represent their interests in particular ways during wildlife management decision-making processes [20], [21]. Given socio-demographics' limited explanatory power for wildlife-related perceptions and behaviors [22], [23], social identities may strengthen predictability of models considering such concepts [24], [25]. Socio-demographic characteristics such as age, education or income may reveal patterns in attitudes but not explain why stakeholders conflict on a fundamental level. Considering SIT within the context of wildlife management and decision-making may produce novel insights upon which to design, implement, and evaluate stakeholder engagement, conflict resolution, and management of HWC [14].

### Social Identity Theory and Wildlife Management

SIT explains how individuals view themselves through their group memberships and the value and meaning attached to that membership [19], [26]. According to SIT, individuals find *ingroups* consisting of like-minded individuals. The individual views himself as a representative of that group and acts according to group expectations and norms [27]. Ingroups are cohesive because of a shared desire for positive social identity (e.g., high self-esteem), which is attained by comparisons of their ingroup to germane outgroups [28]. Comparisons that reveal perceived inequalities in status (e.g., based on socioeconomic levels, power dynamics) result in competition and *ingroup bias*, whereby individuals seek to increase positive ingroup characteristics and negative aspects of outgroups [1], [29]. Ingroup bias also results in the assumption that outgroups are homogenous.

Incorporating SIT into wildlife-related decision-making may advance stakeholder engagement beyond stereotypes, offer another way to understand underlying stakeholder concerns about hunting wolves, and potentially bears implications for resolving human conflict over HWC. Because people tend to underestimate attitudinal heterogeneity within a group (i.e., ingroup bias) and socio-demographics alone may not explain group interactions [13], [30], characterizing social identities may identify stakeholders assumptions about outgroups (i.e., stereotypes) and strengthen understanding of why stakeholder conflict [25], [31], [32]. For example, identity defined by a particular community, interest or lifestyle can predict deeply-held, value-laden perceptions, which people may fight to defend and also ultimately influence behaviors towards wildlife [25], [33], [34]. Documenting attitudinal diversity among identity groups may prove useful in identifying underlying sources of conflict over policy preferences, helping stakeholders relate to opposing group members and guiding communication that addresses underlying concerns [35].

### Intergroup Conflicts over Wolf Hunting in Michigan

Gray wolves were eradicated from the Western Great Lakes region (i.e., Michigan, Minnesota, Wisconsin) except in Northern Minnesota by the 1930s, listed as endangered under the Endangered Species Act in 1973 and naturally emigrated back to Michigan's Upper Peninsula over the past two decades [36]. As of the most recent population estimate from early 2014, Upper Peninsula wolves numbered approximately 636 individuals in 125 packs [37]. In 2012, the United States Congress and Fish and Wildlife Service deemed wolves to be recovered and delisted from the Endangered Species Act. Delisting returned wolf management to state agencies (e.g., Michigan Department of Natural Resources [DNR]). Legislation designating wolves as a game species, lawsuits, and ballot initiatives to overturn legislation followed delisting. Although stakeholder engagement in wolf management has a long history in Michigan [38], these political battles suggest intergroup conflict over hunting wolves has evolved and perhaps intensified over the past few years [39], [40].

In Michigan, some groups (e.g., animal welfare and rights advocates, deer and bear hunters, livestock owners, Native American tribes) have publically taken various positions about hunting wolves and aligned themselves with similarly positioned groups. Although these identity groups may publically present dichotomized pro- or anti-hunting policy positions, their underlying justifications for policy preferences are not necessarily the same. For example, nuanced but important differences of opinion can exist between hunters who hunt deer versus those who hunt bear. Treating identity groups with different concerns about hunting wolves as a single stakeholder group based solely on policy position (e.g., pro or anti wolf hunting) may lead individuals to assume that the opposition holds homogenous attitudes in direct conflict with the individual and his/her identity group [28], [41]. Thus, so-called “pro-wolf hunting” and “anti-wolf hunting” groups seem to make assumptions about each others' positions that are not necessarily accurate [3]. For instance, some stakeholders may believe state wildlife managers hold anti-wolf hunting attitudes while others claim managers favor pro-wolf hunting interests [3]. Another assumption is that people living in a particular geographic region (e.g., rural areas, within wolf range) are uniformly for or against wolf hunting [36], [42]. These assumptions may remain concealed if social identity is not explicitly considered in stakeholder engagement processes and thus the negative aspects of conflict may continue [43], [44]. Given the aforementioned principles of SIT and the context of intergroup conflict over wolf hunting in Michigan, we set and achieved two objectives for this research: (1) characterize concerns about wolf hunting as a management tool, and (2) explore social identities underlying concerns.

## Materials and Methods

### Ethics Statement

Michigan State University's Committee on Research Involving Human Subjects (IRB# x11-1144e) reviewed and approved methods used in this research. Committee-approved informed consent was obtained in written form. Respondents had to first read the informed consent statement, continuing on to the survey was consent to participate in the study.

### Data Archiving

Data are archived at: http://datadryad.org/[DOI will be added after acceptance].

### Sampling Protocol

In March 2013, we used a snowball sampling technique that included two modes, whereby paper and online version of the questionnaire were made available [45]. This approach allowed us to reach diverse individuals within the relevant “issue publics,” in other words, among individuals who are active and aware stakeholders in the issue of wolf management in Michigan [46], [47]. Paper questionnaires were distributed to all interested individuals over 18 years old who attended one of four public meetings conducted by MDNR about wolf hunting (in Ironwood, Marquette, Gaylord and Lansing, MI). Public meeting attendees were encouraged to share the hyperlink to the online survey and a Qualtrics survey option prevented ballot stuffing by preventing duplicate responses from the same IP address. The hyperlink was also posted on the MDNR website. The online survey was live March 13–29, 2013.

### Measurement

Paper and online versions of the survey used identical measures and formats. Five-point Likert-style questions measured twelve concerns regarding hunting wolves as a tool to address conflict. The twelve concerns were originally identified by the Michigan Wolf Advisory Council, a MDNR-initiated group of stakeholder representatives (a group that existed in various forms for years but was required by 2012 Public Act 520, which also designated wolves a game species in Michigan) [48]. Participants were also asked to rank order the twelve concerns according to importance (Table 1). In addition, survey questions using five-point scales measured: (1) caring for wolves (i.e., How much do you care about wolves in Michigan?); (2) fear of wolves (i.e., How much do you believe wolves in the woods can be dangerous to people?); and (3) support for wolf hunting (i.e., How much do you support managing wolf populations in Michigan by hunting?). Care and fear of wolves have been found to be important indicators of support or lack of support, respectively, for Michigan wolf recovery in past studies [42], [49]. State of residence and, if in Michigan, county of residence were also measured. Sex was measured by asking respondents to indicate whether they identified as male or female.

**Table 1:** Concerns about hunting wolves.

Overall Rank	Concern that…	Factor 1	Factor 2	Factor 3
1	…wolf management reduces conflicts with people, livestock, game species, and pets.	0.413	**0.595**	0.068
2	…wolf management is based on scientific research.	**0.746**	0.209	0.125
3	…a wolf population in Michigan be maintained.	**0.590**	−0.038	0.067
4	…harmful effects of wolves on deer populations are avoided.	0.117	**0.763**	−0.025
5	…wolf management is implemented according to the law.	**0.703**	0.188	0.095
6	…MDNR continue to use non-lethal and lethal tools for reducing wolf-related conflicts.	**0.516**	0.125	0.110
7	…the public have enough chances to share their opinions about wolf management.	**0.583**	0.297	−0.048
8	…financial resources for wolf management be used responsibly.	**0.627**	0.253	−0.008
9	…wolf management does not cause harmful changes in wolf pack behavior.	0.021	0.072	**0.870**
10	…wolf management reduces negative attitudes.	0.105	**0.565**	0.490
11	…wolf managers consider differing attitudes about wolf management.	**0.607**	0.061	0.125
12	…hunting could cause more illegal killing of wolves.	**0.492**	−0.344	0.441
% variance		26.8%	13.6%	10.3%

Respondents were asked to rank the importance of the following concerns associated with hunting wolves in Michigan, March 2013. Principal component analysis of concerns was conducted and concerns were assigned to the factor in which they load highest and ≧0.45.

### Data Analysis

Paper and web-based survey responses were pooled into a single dataset and duplicate responses (subsequently dated IP addresses) were deleted [45]. We also searched for duplicate cases based on sociodemographic characteristics in the chance that individuals filled out both a paper and online survey. We used a general linear model (Type III Sum of Squares) to explore whether care for, fear of wolves and region of residence (i.e., Michigan vs. non-Michigan states) were related to concerns about hunting wolves [50]. We used principal components analysis with a Varimax rotation and eigenvalues >1 to analyze how concerns related to each other and used support for hunting as the selection variable [50]. Concerns were assigned to the factor in which they load highest and ≧0.45. We used eigenvalue, factor loading and scree test to determine the number of factors included and factors that explained <10% variation were not included [51]. All analyses were conducted in SPSS 19.0 (SPSS Inc., Chicago, USA).

## Results

### Sample Characteristics

A total of 676 respondents completed our survey and 10 duplicate electronic responses were deleted for a usable sample of 666. Michigan residents comprised the majority (n = 625, 94%) of respondents. Upper Peninsula residents were overrepresented (n = 319, 48%) in the sample compared to their relative proportion in the statewide population (n = 311361, 3%) [52]. Respondents from 21 other states completed the survey (Alabama, California, Colorado, Connecticut, Delaware, Florida, Georgia, Idaho, Illinois, Indiana, Kentucky, Massachusetts, Minnesota, Montana, New York, Ohio, Oklahoma, Oregon, Texas, Washington D.C., Wisconsin). Self-identified hunters (n = 349, 52%) and trappers (n = 95, 14%) were overrepresented in our sample compared to published recreational participation records (n = 795535, 8% for hunters and n = 10241, 0.1% for trappers in MI, respectively)[53], [54]. Internet responses consisted of 48% (n = 322) of the total response. Of the total sample, 350 respondents (52.6%) had attended one of the four MDNR-led public meetings. Twenty-two percent (n = 146) of respondents were female and 78% percent (n = 520) were male.

Of the 12 concerns presented, the top-ranked three concerns associated with hunting as a tool to manage wolves were about: (1) managing conflicts with wolves, (2) use of science in decision-making, (3) and maintaining a wolf population (Table 1). Support for hunting was bimodal with extremes dominating the responses; respondents agreed (n = 328, 63%) or disagreed (n = 166, 32%). When asked how much they cared about wolves, responses were skewed toward caring (n = 413, 80%; versus not caring, n = 69, 13%). Fear of wolves was balanced with 38% (n = 195) disagreeing that they believe wolves are dangerous and 47% (n = 243) agreeing with the statement.

### Concerns about Wolf Hunting as a Management Tool

Principle components analysis revealed (KMO = 0.821, Barlett's X^2^ = 662.394, df = 66, p<0.01) that all 12 concerns loaded significantly (≧0.45) on three factors that explained 50.7% of total variance (Table 1). Explaining 26.8% of variance, Factor 1 consisted of 8 concerns that: (1) wolf management is based on scientific research, (2) wolf managers consider differing attitudes about wolf management, (3) the public have enough chances to share their opinions about wolf management, (4) wolf management is implemented according to the law, (5) financial resources for wolf management be used responsibly, (6) a wolf population in Michigan be maintained, (7) MDNR continue to use non-lethal and lethal tools for reducing wolf-related conflicts and (8) hunting could cause more illegal killing of wolves. Factor 2 explained 13.6% of variance and consisted of three concerns that: (1) wolf management may reduce negative attitudes, (2) wolf management may reduce conflicts with people, livestock, game species, and pets and (3) harmful effects of wolves on deer populations are avoided. Factor 3 consisted of only one concern that wolf management does not cause harmful changes in wolf pack behavior yet explained 10.3% of variance. We kept this third factor in the analysis based on eigenvalue, factor loading and scree test [51]. We chose not to assign labels to factors, but suggest the grouping of factors revealed by this analysis may correspond to concerns emphasized by identity group.

### Effects on Concerns about Wolf Hunting

**Figure 1:**
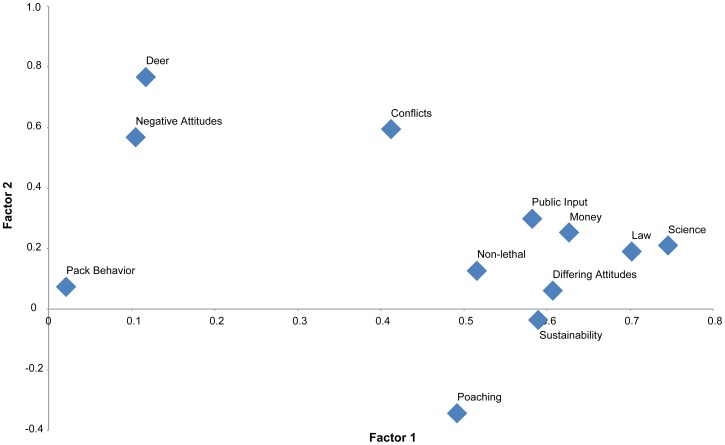
Principal components analysis revealed 12 concerns loaded significantly (≥0.45) on three factors (KMO = 0.821, Barlett's X^2^ = 662.394, df = 66, p<0.01), explaining 50.7% of total variance. Factor 1 explained 26.8% of variance and consisted of 8 concerns. Factor 2 explained 13.6% of variance and consisted of 3 concerns. Concerns about changes to pack behavior made up Factor 3, which explained 10.3% of variance.

General linear model analysis revealed care was significantly and positively correlated with concerns about hunting wolves (Sum of Squares = 180.140, df = 83, R^2^ = 2.170, F = 1.504, p<0.01; Figure 1). In other words, the more a respondent cared about wolves the more they agreed with concerns about hunting as a tool to address conflict. Fear was significantly and negatively correlated with concerns about hunting wolves (Sum of Squares = 298.190, df = 83, R^2^ = 3.593 F = 1.916, p<0.01); more fearful respondents were more likely to disagree with concerns. Among respondents, region of residence was not significantly related to concerns (Sum of Squares = 2.642, df = 82, R^2^ = 0.032, F = 0.644, p>0.01).

## Discussion

Our first objective was to use principles from SIT to help characterize concerns about wolf hunting as a management tool; results suggest identity groups may crystallize over justifications for support or opposition to hunting. Our second objective was to explore social identity-related factors underlying concerns about wolf hunting. Results revealed that among study participants wolf-related identities centered on care and fear of wolves and not geography, challenging assumptions that regional and urban-rural differences drive patterns in policy preferences for wildlife management.

Findings suggest social identity based on positive (e.g., care) or negative (e.g. fear) attitudes toward wolves, not region, may be important factors driving public perceptions about wolf hunting [25], [30], [34], [55]. Although work measuring sense of place has contributed useful insight into human dimensions of wildlife [56]–[58], stakeholders may organize and interact less on local place-based scales than on more global scales due to the ease of contemporary communication and associated networks (e.g., mobile phones, social media) [59], [60]. Indeed, social identity research has demonstrated group identification can be strong despite geographic distance and has been used to understand globalized ethnic conflict whereby individuals identify with a distant, threatened identity group [61]. Similarly, sense of self, in relation to wolf management and perhaps many other HWC situations, may be driven less by place in the physical geographic sense and more in the social geography sense. Future research may test these assumptions in other HWC contexts. If born out empirically and in other contexts, inequalities among groups across spatially and temporally large scales may play an important role in apparently local disputes over wildlife and other natural resources.

Our results reaffirm literature suggesting social identities among stakeholders highly involved in wolf management are polarized (i.e., between those who care and those who fear wolves) and concerns about hunting wolves are easily dichotomized (into the two predominant Factors 1 and 2)[3], [62]. The polarization of wolf-related stakeholders may not seem like new information; indeed, wolf management has been divisive for decades. We believe our results are meaningful because they provide empirical evidence that polarization still exists. Given the many policy changes and attempts at conflict resolution that have occurred since prior attitudinal research was conducted, such findings are important for informing improved stakeholder engagement [63].

Although positions and interests related to wildlife management can be nuanced and diverse, the dichotomy among study participants vis-à-vis current wolf management may be the result of group reactions to perceived status threats [64]. Hunters may feel their traditions and culture are less accepted in mainstream society, evidenced by decreases in hunter retention and recruitment and ballot initiatives to ban certain practices [65], [66]. Animal welfare and rights advocates may also see threats to group status and thus fight to have their interests represented by agencies that have historically focused on hunter interests [67]. Interestingly, the study participants did not rank concerns about public perceptions and input (e.g., considering different attitudes, having enough chances for public input, reducing negative attitudes) very high. One might expect the public to value chances for input and rank such considerations higher than we observed. Perhaps low ranking of these concerns indicates issues that management agencies have successfully addressed or where there is little disagreement between groups. Alternatively, if stakeholders believe decision-makers are biased, they may not see value in participating in public input processes and may fear their participation would validate a process that promotes the opposing groups' desired outcome [68]. Ultimately, better understanding about the causes and consequences of polarization would make for fruitful research avenues.

SIT provides insight about how to cope with the dichotomies for communication within groups and cooperation among groups such as those found in among our study participants. First, communication geared toward each identity group that addresses their specific concerns may be more persuasive and effective than messages targeting stakeholders generally. Stakeholder engagement that fails to address the unique concerns of a particular stakeholder may result in magnified negative effects such as lawsuits and noncompliance [69]. Identity-specific communication delivered separately to each identity group may help build trust between agencies and public stakeholders [35]. Communication that builds trust, empowers stakeholders equally and contributes to a sense of procedural justice may increase support for decision-makers and processes regardless of decision outcome [70], [71].

Second, SIT tells us simultaneously encouraging “care” for wolves while decreasing “fear” as measured by our survey instrument may help usher identity groups toward common understanding and greater agreement about management strategies. Because our results suggest that fear influences management acceptability, mitigating perceived risk may be an especially effective tool to address conflict over HWC. Strategic risk communication may help decrease “fear of wolves” by emphasizing wolf-related benefits [72], [73] or highlight concerns of one group to the other group. For example, risk messages addressing concerns about wolves' impacts on deer populations could be emphasized to groups opposed to wolf hunting or the effectiveness of nonlethal techniques could be communicated to groups supportive of wolf hunting. Furthermore, making salient the higher order organizational affiliation (e.g., advisory council) or superordinate goals (e.g., maintaining populations) over personal identities may encourage intergroup cooperation [20], [29], [74]. Encouraging cooperation may be accomplished by priming individuals to think in terms of “we” and not “I,” assigning common tasks or ensuring that external cues (e.g., identity-typical clothing, colloquial language) do not reinforce group differences [74]. In theory, communication techniques that succeed in blurring the lines between smaller, specific group identities and leveling status inequalities between these groups may create interdependence between personal and collective objectives, which may increase cooperation [75]. In practice however, managers may need to use caution so as not to appear to be deliberately sending different messages to different groups because it might give the impression of dishonest or one-sided messaging; principles from effective and ethical risk communication may be useful here [76].

The salience of a broader, inclusive group identification can also be increased by emphasizing the distinctive qualities of that group and areas where group standing can be improved [75]. Given the challenges of HWC stakeholder engagement, stakeholders may be readily motivated by the goals of improving upon prior conflict and providing a successful model for future conflict mitigation. Although some studies have found that common identity can be primed and cooperation achieved simply by emphasizing inclusive language (e.g., literally using the word “we”) or other cues (e.g., avoiding uniforms that emphasize hierarchal differences)[74], we recognize that encouraging cooperation among polarized stakeholders is no small feat. Overcoming years of historical conflict cannot be accomplished with a single workshop or public meeting; SIT advises priming inclusive group salience must be continuous to be sustained through changing circumstances and result in truly interpersonal interactions and mutual understanding [64], [75].

How the case of wolf management evolves in Michigan has implications for large carnivore management in diverse contexts and other regions [77]. Conflict among and between groups over management objectives is likely to play out repeatedly as other large carnivores, such as Eurasian brown bears (*Ursus arctos arctos*), return to historic ranges [78]. Anticipating potential tension between competing identity groups, managers might develop identity-specific (versus sociodemographic-specific) communication and encourage broader interests to prevent perceptions of inequality before they form. Future research could use confirmatory factor analysis to further explore these preliminary results regarding relationships of care for and fear of wolves to concerns about hunting wolves. Additionally, other factors beyond care for and fear of wolves may be important and should be explored. Interventions aimed at broadening concerns, increasing care for wolves and decreasing fear of wolves may be useful in improving stakeholder engagement and efficacy of communication.

## References

[1] Labianca G, Brass DJ, Gray B (1998) Social networks and perceptions of intergroup conflict: The role of negative relationships and third parties. Acad Manag J 41:55–67.

[2] Triezenberg HA, Knuth BA, Yuan C (2011) Evolution of public issues in wildlife management: how social networks and issue framing change though time. Hum Dimens Wildl 16:381–396.

[3] Lute ML, Gore ML (2014) Stewardship as a path to cooperation? Exploring the role of identity in intergroup conflict among Michigan wolf stakeholders. Hum Dimens Wildl 19:267–279.

[4] Gore ML, Ratsimbazafy J, Lute ML (2013) Rethinking corruption in conservation crime: insights from Madagascar. Conserv Lett 6:430–438 Available: http://doi.wiley.com/10.1111/conl.12032 Accessed 28 August 2013.

[5] Keane A, Jones JPG, Edwards-Jones G, Milner-Gulland EJ (2008) The sleeping policeman: understanding issues of enforcement and compliance in conservation. Anim Conserv 11:75–82 Available: http://doi.wiley.com/10.1111/j.1469-1795.2008.00170.x Accessed 4 June 2013.

[6] Treves A, Naughton-Treves L, Shelley V (2013) Longitudinal Analysis of Attitudes Toward Wolves. Conserv Biol 27:315–323 Available: http://www.ncbi.nlm.nih.gov/pubmed/23293913 Accessed 11 February 2013. 23293913 10.1111/cobi.12009

[7] Bruskotter JT, Enzler SA, Treves A (2011) Rescuing Wolves from Politics: Wildlife as a Public Trust Resource. Science (80-) 333:1828–1829. 10.1126/science.1207803 21960614

[8] Jacobson CA, Organ JF, Decker DJ, Batcheller GR, Carpenter L (2010) A Conservation Institution for the 21st Century: Implications for State Wildlife Agencies. J Wildl Manage 74:203–209 Available: http://www.bioone.org/doi/abs/10.2193/2008-485 Accessed 5 June 2013.

[9] Leong KM, Mccomas KA, Decker DJ (2007) Matching the Forum to the Fuss: Using Coorientation Contexts to Address the Paradox of Public Resource Management. Environ Pract 3:195–205.

[10] Warriner GK, Madden JJ, Lukasik L, McSpurren K (1996) Public Participation in Watershed Management: a Comparative Analysis. Can Water Resour J 21:253–273 Available: http://www.tandfonline.com/doi/abs/10.4296/cwrj2103253 Accessed 20 December 2013.

[11] Kellon D, Arvai J (2011) Five propositions for improving decision making about the environment in developing communities: Insights from the decision sciences. J Environ Manage 92:363–371 Available: http://www.sciencedirect.com/science/article/pii/S0301479710003385. 21035941 10.1016/j.jenvman.2010.10.010

[12] Bright AD, Manfredo MJ, Fulton DC (2000) Segmenting the Public: An Application of Value Orientations to Wildlife Planning in Colorado. Wildl Soc Bull 28:218–226 Available: http://www.jstor.org/stable/4617305.

[13] Glikman J, Bath A, Vaske J (2010) Segmenting Normative Beliefs Regarding Wolf Management in Central Italy. Hum Dimens Wildl 15:347–358 Available: http://www.tandfonline.com/doi/abs/10.1080/10871209.2010.505598 Accessed 16 December 2013.

[14] Madden F (2004) Creating Coexistence between Humans and Wildlife: Global Perspectives on Local Efforts to Address Human–Wildlife Conflict. Hum Dimens Wildl 9:247–257 Available: http://www.tandfonline.com/doi/abs/10.1080/10871200490505675 Accessed 20 December 2013.

[15] Treves A, Wallace RB, Naughton-Treves L, Morales A (2006) Co-Managing Human–Wildlife Conflicts: A Review. Hum Dimens Wildl 11:383–396 Available: http://www.tandfonline.com/doi/abs/10.1080/10871200600984265 Accessed 29 May 2013.

[16] Treves A, Wallace RB, White S (2009) Participatory planning of interventions to mitigate human-wildlife conflicts. Conserv Biol 23:1577–1587 Available: http://www.ncbi.nlm.nih.gov/pubmed/19459896 Accessed 25 October 2013. 19459896 10.1111/j.1523-1739.2009.01242.x

[17] Webler R, Tuler S, Krueger T (2001) What Is a Good Public Participation Process? Five Perspectives from the Public. Environ Manage 27:435–450. 11148768 10.1007/s002670010160

[18] Hornsey MJ (2008) Social Identity Theory and Self-categorization Theory: A Historical Review. Soc Personal Psychol Compass 2:204–222 Available: http://doi.wiley.com/10.1111/j.1751-9004.2007.00066.x.

[19] Tajfel H, Turner JC (1979) An integrative theory of intergroup conflict. In: Austin WG, Worchel S, editors. The Social Psychology of Intergroup Relations. Monterey, CA: Brooks/Cole. pp.33–47.

[20] Ashforth BE, Johnson SA (2001) Which hat to wear? The relative salience of multiple identities in organizational contexts. In: Hogg MA, Terry DJeditors. Social identity processes in organizational contexts. Philadelphia, PA: Psychology Press. pp.31–48.

[21] Hogg MA, Fielding KS, Johnson D, Masser B, Russell E, et al (2006) Demographic category membership and leadership in small groups: A social identity analysis. Leadersh Q 17:335–350 Available: http://linkinghub.elsevier.com/retrieve/pii/S1048984306000361 Accessed 11 June 2013.

[22] Enck JW, Brown TL (2002) New Yorkers' attitudes toward restoring wolves to the Adirondack Park. Wildl Soc Bull 30:16–28.

[23] Vaske JJ, Donnelly MP, Williams DR, Jonker S (2001) Demographic Influences on Environmental Value Orientations and Normative Beliefs About National Forest Management. Soc Nat Resour 14:761–776.

[24] Manfredo M (2008) Who Cares About Wildlife? New York: Springer.

[25] Naughton-Treves L, Grossberg R, Treves A (2003) Paying for Tolerance: Rural Citizens' Attitudes toward Wolf Depredation and Compensation. Conserv Biol 17:1500–1511 Available: http://doi.wiley.com/10.1111/j.1523-1739.2003.00060.x.

[26] Tajfel H (1982) Social Psychology of Intergroup Relations. Annu Rev Psychol 33:1–39.

[27] Jetten J, Postmes T, McAuliffe BJ (2002) We're all individuals?: Group norms of individualism and collectivism, levels of identification and identity threat. Eur J Soc Psychol 32:189–207 Available: http://doi.wiley.com/10.1002/ejsp.65 Accessed 31 January 2013.

[28] Brown R (2000) Agenda 2000 Social Identity Theory: past achievements, current problems and future challenges. Eur J Soc Psychol 30:634–667.

[29] Sherif M (1966) Group Conflict and Cooperation: Their Social Psychology. London: Routledge & Kegan Paul Ltd.

[30] Sponarski CC, Semeniuk C, Glikman J a, Bath AJ, Musiani M (2013) Heterogeneity among Rural Resident Attitudes Toward Wolves. Hum Dimens Wildl 18:239–248 Available: http://www.tandfonline.com/doi/abs/10.1080/10871209.2013.792022 Accessed 18 September 2013.

[31] Scales IR (2012) Lost in translation: conflicting views of deforestation, land use and identity in western Madagascar. Geogr J 178:67–79 Available: http://doi.wiley.com/10.1111/j.1475-4959.2011.00432.x Accessed 6 June 2013. 22413174 10.1111/j.1475-4959.2011.00432.x

[32] Knezevic I (2009) Hunting and Environmentalism: Conflict or Misperceptions. Hum Dimens Wildl 14:12–20 Available: http://www.tandfonline.com/doi/abs/10.1080/10871200802562372 Accessed 4 June 2013.

[33] Kaltenborn BP, Bjerke T, Vitterso J (1999) Attitudes toward Large Carnivores among Sheep Farmers, Wildlife Managers, and Research Biologists in Norway. Hum Dimens Wildl 4:37–41.

[34] Skogen K, Krange O (2003) A wolf at the gate: The anti-carnivore alliance and the symbolic construction of community. Sociol Ruralis 43: 309–325. Available: <Go to ISI>://000184840800008.

[35] Kahan D (2010) Fixing the communications failure. Nature 463:296–297 Available: http://www.ncbi.nlm.nih.gov/pubmed/20090734. 20090734 10.1038/463296a

[36] Beyer D, Hogrefe T, Peyton RB, Bull P, Burroughs JP, et al**.** (2006) Review of social and biological science relevant to wolf management in Michigan. Lansing, Michigan.

[37] Wolf Biology and Identification (n.d.) Michigan Dep Nat Resour. Available: http://www.michigan.gov/dnr/0,4570,7-153-10370_12145_12205_63607_63608-292026-,00.html. Accessed 31 January 2014.

[38] Lute ML (2013) Human Dimensions of Wolf Management in Michigan: A review of literature. Lansing, Michigan.

[39] Oosting J (2013) New Michigan group seeks to protect future wolf hunts with citizen-initiated legislation. MLive. Available: http://www.mlive.com/politics/index.ssf/2013/11/new_michigan_group_seeks_to_pr.html.

[40] Press A (2013) Wolf hunt referendum to go on 2014 Michigan ballot. Detroit Free Press.

[41] Abrams D, Wetherell M, Cochrane S, Hogg MA, Turner JC (1990) Knowing what to think by knowing who you are: self-categorization and the nature of norm formation, conformity and group polarization. Br J Soc Psychol 29:97–119 Available: http://www.ncbi.nlm.nih.gov/pubmed/2372667. 2372667 10.1111/j.2044-8309.1990.tb00892.x

[42] Mertig AG (2004) Attitudes about wolves in Michigan, 2002. Final report to Michigan Department of Natural Resources. East Lansing, Michigan.

[43] Dickman AJ (2010) Complexities of conflict: The importance of considering social factors for effectively resolving human-wildlife conflict. Anim Conserv 13:458–466 Available: http://doi.wiley.com/10.1111/j.1469-1795.2010.00368.x Accessed 6 August 2013.

[44] Nie M (2003) Drivers of natural resource-based political conflict. Policy Sci 36:307–341.

[45] Dillman D, Smyth J, Christian L (2009) Internet, mail, and mixed-mode surveys: the tailored design method. 3rd ed. Hoboken: Wiley.

[46] Grunig J (1979) A new measure of public opinions on corporate social responsibility. Acad Manag J 22:738–764.

[47] Krosnick JA (1990) Government policy and citizen passion: A study of issue publics in contemporary America. Polit Behav 12:59–92.

[48] Gore ML, Lute ML (2013) Michigan Wolf Forum December 2012. Lansing, Michigan.

[49] Kellert SR (1990) Public Attitudes and Beliefs About the Wolf and its Restoration in Michigan.

[50] Vaske JJ (2008) Survey research and analysis: applications in parks, recreation and human dimensions. State College, PA: Venture Publishing.

[51] Costello AB, Osborne JW (2005) Best Practices in Exploratory Factor Analysis: Four Recommendations for Getting the Most From Your Analysis. Pract Assessment, Res Eval 10:1–9 Available: http://pareonline.net/getvn.asp?v=10&n=7.

[52] Census 2010 (2010) Available: http://factfinder2.census.gov.

[53] Michigan Department of Natural Resources: Economic Impact (n.d.). Michigan Dep Nat Resour. Available: http://www.michigan.gov/dnr/0,4570,7-153-10366-121641-,00.html. Accessed 31 January 2014.

[54] Frawley BJ (2013) 2012 Michigan Furbearer Harvest Survey. Lansing, Michigan.

[55] Skogen K (2001) Who's afraid of the big, bad wolf? Young people's responses to the conflicts over large carnivores in eastern Norway. Rural Sociol 66: 203–226. Available: <Go to ISI>://000169215800003.

[56] Cantrill J (2011) The Role of a Sense of Self-in-Place and Risk Amplification in Promoting the Conservation of Wildlife. Hum Dimens Wildl 16:73–86 Available: http://www.tandfonline.com/doi/abs/10.1080/10871209.2011.542555 Accessed 16 November 2013.

[57] Grey ME (2012) Interpreting the significance of protected areas: A case study of how recreationists value the Craigieburn and Castle Hill conservation areas, Canterbury, New Zealand Lincoln University.

[58] Jorgensen BS, Stedman RC (2001) Sense of Place As an Attitude: Lakeshore Owners Attitudes Toward Their Properties. J Environ Psychol 21:233–248 Available: http://linkinghub.elsevier.com/retrieve/pii/S0272494401902269 Accessed 11 November 2013.

[59] Halpern D, Gibbs J (2013) Social media as a catalyst for online deliberation? Exploring the affordances of Facebook and YouTube for political expression. Comput Human Behav 29:1159–1168 Available: http://linkinghub.elsevier.com/retrieve/pii/S0747563212002762 Accessed 21 January 2014.

[60] Rhue L, Sundararajan A (2014) Digital access, political networks and the diffusion of democracy. Soc Networks 36:40–53 Available: http://linkinghub.elsevier.com/retrieve/pii/S0378873312000524 Accessed 23 January 2014.

[61] Spears R (2011) Group Identities: The Social Identity Perspective. In: Schwartz SJ, Luyckx K, Vignoles VLeditors. Handbook of Identity Theory and Research. New York, NY. pp.201–224.

[62] Peyton R, Bull P, Holsman R (2007) Measuring the social carrying capacity for gray wolves in Michigan. Lansing, Michigan.

[63] Olson ER, Stenglein JL, Shelley V, Rissman AR, Browne-Nuñez C, et al**.** (2014) Pendulum swings in wolf management led to conflict, illegal kills, and a legislated wolf hunt. Conserv Lett 0: n/a–n/a. Available: http://doi.wiley.com/10.1111/conl.12141. Accessed 3 October 2014.

[64] Jackson JW (1993) Realistic group conflict theory: A review and evalution of the theoretical and empirical literature. Psychol Rec 43:395–413.

[65] Williamson SJ (2008) Origins, history, and current use of ballot initiatives in wildlife management. Hum Dimens Wildl Manag 3:51–59.

[66] Ryan EL, Shaw B (2011) Improving Hunter Recruitment and Retention. Hum Dimens Wildl 16:311–317 Available: http://www.tandfonline.com/doi/abs/10.1080/10871209.2011.559530 Accessed 24 February 2014.

[67] Nie MA (2002) Wolf Recovery and Management as Value-based Political Conflict. Ethics, Place Environ 5:65–71 doi:10.1080/1366879022014646

[68] Tyler TR (2000) Social Justice: Outcome and Procedure. Int J Psychol 35:117–125 Available: http://doi.wiley.com/10.1080/002075900399411.

[69] Minnis DL (1998) Wildlife Policy-Making by the Electorate: An Overview of Citizen-Sponsored Ballot Measures on Hunting and Trapping. Wildl Soc Bull 26:75–83 Available: http://www.jstor.org/stable/3783804.

[70] Tyler TR (1994) Psychological Models of the Justice Motive: Antecedents of Distributive and Procedural Justice. J Pers Soc Psychol 67:850–863.

[71] Winter S, May P (2001) Motivation for compliance with environmental regulations. J Policy Anal Manag 20:675–698.

[72] Slagle KM, Bruskotter JT, Wilson RS (2012) The Role of Affect in Public Support and Opposition to Wolf Management. Hum Dimens Wildl 17:44–57 Available: http://www.tandfonline.com/doi/abs/10.1080/10871209.2012.633237 Accessed 22 January 2014.

[73] Zajac RM, Bruskotter JT, Wilson RS, Prange S, Dimensions H (2012) Learning to live with black bears: A psychological model of acceptance. J Wildl Manage 76:1331–1340 Available: http://doi.wiley.com/10.1002/jwmg.398 Accessed 31 May 2013.

[74] Ford J, O′Hare D, Henderson R (2012) Putting the “We” Into Teamwork: Effects of Priming Personal or Social Identity on Flight Attendants' Perceptions of Teamwork and Communication. Hum Factors J Hum Factors Ergon Soc 55:499–508 Available: http://hfs.sagepub.com/cgi/doi/10.1177/0018720812465311 Accessed 10 August 2013. 10.1177/0018720812465311 23829025

[75] Ellemers N, Gilder D De, Haslam SA, Gilder DDE (2013) Motivating individuals and groups at work: A social identity perspective on leadership and group performance. Acad Manag Rev 29:459–478.

[76] Jurin RR, Roush D, Danter J (2010) Environmental Communication. 2nd ed. Dordrecht: Springer.

[77] Treves A (2009) Hunting for large carnivore conservation. J Appl Ecol 46:1350–1356 Available: http://doi.wiley.com/10.1111/j.1365-2664.2009.01729.x Accessed 14 October 2012.

[78] Rigg R, Find′o S, Wechselberger M, Gorman ML, Sillero-Zubiri C, et al (2011) Mitigating carnivore–livestock conflict in Europe: lessons from Slovakia. Oryx 45:272–280 Available: http://www.journals.cambridge.org/abstract_S0030605310000074 Accessed 18 September 2013.

